# Synthesis of lanthanide tag and experimental studies on paramagnetically induced residual dipolar couplings

**DOI:** 10.1186/s13065-022-00847-5

**Published:** 2022-07-21

**Authors:** Ali Yassin, Bilal Nehmeh, Sally El Kantar, Yara Al Kazzaz, Elias Akoury

**Affiliations:** 1 Department of Natural Sciences, School of Arts and Sciences, Lebanese American University, Beirut, 1102-2801 Lebanon; 2 Inorganic and Organometallic Coordination Chemistry Laboratory, Faculty of Science, LCIO, Lebanese University, Beirut, Lebanon; 3 TIMR (Integrated Transformations of Renewable Matter), Centre de Recherche Royallieu, Université de Technologie de Compiègne, ESCOM, CS 60 319, 60203 Compiègne Cedex, France; 4 Department of Chemistry, Faculty of Chemistry and Pharmacy, Ludwig Maximilian University, 81377 Munich, Germany

**Keywords:** Paramagnetic lanthanide tag, NMR spectroscopy, Residual dipolar coupling, Sulfur chemistry, Spin quantum mechanics

## Abstract

Nuclear Magnetic Resonance (NMR) spectroscopy is an indispensable technique for the structure elucidation of molecules and determination of their characteristic interactions. Residual Dipolar Coupling (RDC) is an NMR parameter that provides global orientation information of molecules but necessitates the use of an anisotropic orientation medium for the partial alignment of the target molecule with respect to the magnetic field. Importantly, anisotropic paramagnetic tags have been successful as orienting media in biomolecular NMR applications but their use in small organic molecules remains imperfect due to challenges in designing functional lanthanide complexes with varying degrees of bonding in the Ln(III) inner coordination sphere. In this study, we propose a strategy for the synthesis of the lanthanide tag 4-mercaptomethylpyridine-2,6-dicarboxylic acid, 4-MMDPA and the measurement of RDCs in a target molecule using several paramagnetic lanthanide complexes.

**Graphical Abstract:**

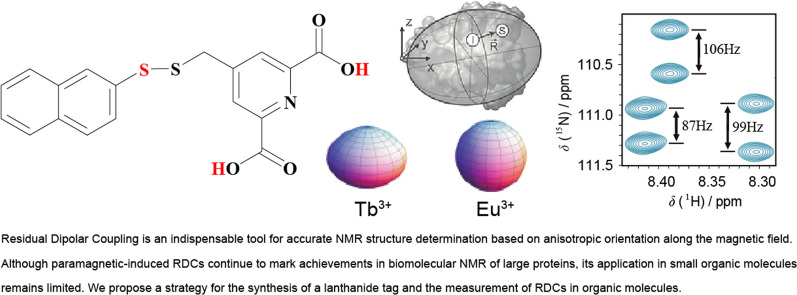

**Supplementary Information:**

The online version contains supplementary material available at 10.1186/s13065-022-00847-5.

## Introduction

**Fig. 1:**
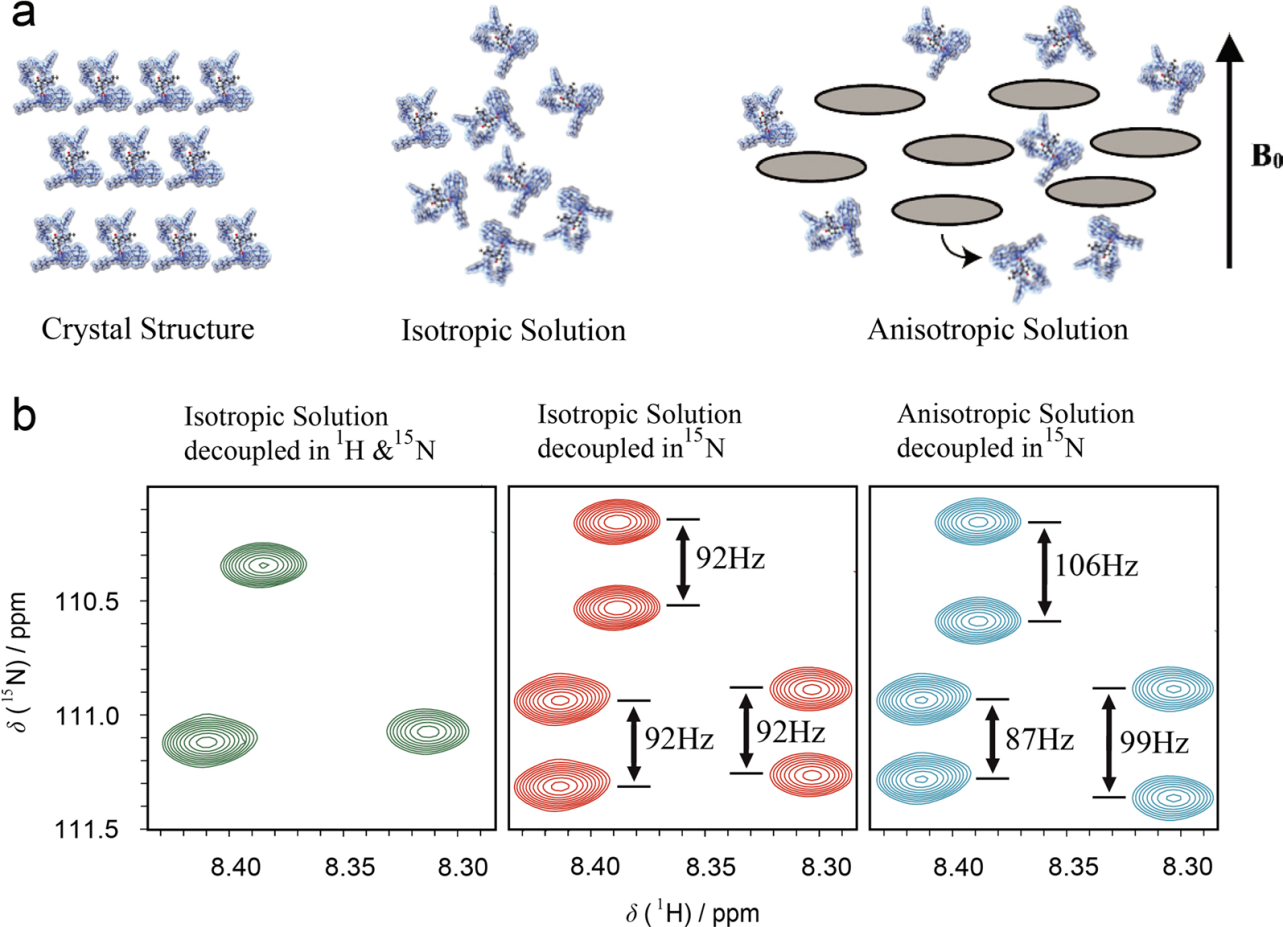
Residual Dipolar Couplings in NMR Spectroscopy. **a** The behavior of a molecule with the magnetic field B_0_ depends on physical properties. In its crystal form, the dipolar couplings are too larger, in an isotropic solution the dipolar interaction average to zero due to tumbling and no coupling is observed, whereas in an anisotropic solution with a partially oriented medium, small residual dipolar couplings are visible. **b** 2D ^1^H-^15^ N HSQC spectra of a protein where each peak corresponds to the N–H resonance of an amino acid of the backbone. The spectra in green and red correspond to an isotropic solution decoupled in both ^1^H and ^15^ N dimensions or only ^15^ N, respectively. The latter spectrum shows the same ^15^ N-^1^H splitting for every amino acid peak and represents ^15^ N-^1^H one-bond scalar coupling. The blue spectrum corresponds to the anisotropic solution decoupled in ^15^ N Dimension. The partial orientation allows observation of ^15^ N-^1^H splitting and represents ^15^ N-^1^H one-bond scalar coupling in addition to the residual dipolar coupling RDC, the total coupling

Residual dipolar couplings (RDCs) have become a fundamental approach in structural determination of proteins, nucleic acids, and carbohydrates. RDCs are spectroscopic interactions that appear in high resolution Nuclear Magnetic Resonance (NMR) spectroscopy. Although it was originally discovered for small molecules in liquid crystal solvents, [1] the RDC spectra were too complex for practical use in structure determination. However, the discovery of weak orienting media in aqueous solutions led to a boost in their application in biomolecular analysis. RDCs can provide long-range angular and distance restraints to solve structural ambiguities, an advantage over the conventional Nuclear Overhauser Effect (NOE) analysis, which is limited to 5 Å radius [2]. The behavior of a molecule with the magnetic field differs largely if it is in crystal form, in isotropic solution or in anisotropic solution with a partially oriented medium (Fig. 1a). RDCs are orientation-dependent interactions that are observed in an anisotropic environment provided by suitable alignment media. This anisotropy allows extraction of angular information relative to an external reference, determination of conformations and configurations of molecules and the distinguish of enantiomers [3]. While dipolar coupling interactions are dominant in solid-state NMR, they are averaged to zero in liquid-state NMR due to isotropic uniform distribution of the orientations based on the *Rotational Brownian Diffusion*. Consequently, a wealth of structural insights is lost once the dipolar couplings vanish. Nevertheless, once displayed in a homogenous magnetic field together with an alignment media, the molecule adapts a preferred orientation that favors anisotropy. This allows the anisotropic magnetic interactions to become observable [4].

The advancement of RDCs in protein NMR spectroscopy has become a routine tool for accurate protein solution structure determination [5–7]. RDCs provide orientation information of magnetic dipole–dipole interaction vectors and its measurement requires a nonisotropic orientation, through a direct or indirect magnetic field alignment, of the protein in solution. The conventional pulse sequences used for coupling measurements, *HMQC and HSQC* experiments, can be recorded without heteronuclear decoupling during acquisition. To decipher RDCs in protein NMR spectroscopy, 2D ^1^H-^15^ N HSQC spectra of isotropic or anisotropic solution only decoupled in ^15^ N dimension are usually measured and analyzed (Fig. 1b). For instance, a regular HSQC spectrum of an isotropic solution decoupled in both dimensions shows no observed ^1^H-^15^ N splitting. On the contrary, when the HSQC spectrum of the same isotropic solution is recorded while decoupling in ^15^ N dimension only, the splitting becomes observable and is equal to a one-bond scalar coupling ^1^J_NH_, baring a negative value between 92 and 95 Hz. The same ^15^ N decoupled HSQC spectrum but of a partially oriented sample shows positive and negative *RDC* values in addition to the observable ^1^H-^15^ N splitting of the one–bond scalar coupling. This observation demonstrates the benefits and advantages of using an orienting medium to induce partial molecular alignment for target molecules as a method to permit accurate molecular conformation and configuration information in solution NMR via the measurement of dipolar couplings, and consequently internuclear distances.

The most common orienting media used for partially aligning a molecule are liquid crystals, strain-induced alignment gels (SAGs), and anisotropic paramagnetic tags. The application of liquid crystals is limited to small symmetric molecules since they induce high degree of order and consequently large dipolar couplings [4]. On the other hand, the alignment stretch of SAG is solely determined by mechanical stretching, and its use is limited due to the lengthy preparation time for solutes to diffuse into the polymer gel [8]. An alternative alignment method, the paramagnetic lanthanide tag, has implemented considerable advances in the field of biomolecular NMR of large proteins, oligonucleotides and drug discovery [9–12]. Mainly, this involves a lanthanide ion that orients molecules by the anisotropy of the paramagnetic susceptibility [13]. Paramagnetic centers have the advantage of providing long-range structural restraints and accordingly ingenious applications in structure determination of proteins, ligand–protein interactions and complex dynamics [14, 15]. So far, the use of lanthanide tags has not been applied to small organic molecules due to the broadening effects in spectral lines of neighboring nuclei covalently attached to the paramagnetic ions [2]. If the intrinsic binding affinity of a paramagnetic tag is high enough, paramagnetically induced RDCs will be observed due to the alignment of the molecule with the magnetic field; in addition to the pseudocontact chemical shift (PCS) caused by the shift tensor anisotropy of the paramagnetic tag. By direct attachment of the lanthanide, the relative mobility of the tag versus the molecule will be minimized, whereas the internal mobility of the small molecule will still exist. Starting from ab initio calculation from the structure of the local complex, the alignment tensor will determine the induced RDCs and PCSs from both a paramagnetic and diamagnetic contribution to the magnetic susceptibility anisotropy. Once the alignment tensor that is attached to the moiety of the molecule bound to the tag has been determined, mobility can be considered much simpler than in case of external alignment, since the induced alignment tensor is fixed.

In this study, we describe the strategy of synthesizing and applying a dipicolinic acid known for its lanthanide tagging of proteins using a single cysteine residue [16]. The tag 4-mercaptomethyl-dipicolinic acid (4-MMDPA) coordinates metal ions in a non-chiral fashion and can be readily attached to a cysteine thiol group via disulfide bridging. Sulfur chemistry has proven to be a powerful tool in synthetic strategies due to its divergent functions and potencies in different oxidative states which have led to a rich chemistry in organic synthesis [17–19]. Sulfide bonds have been reported to create molecular junctions in thiophene-based molecules for example, [20] while thiols were used to functionalize gold nanoparticles with electro-polymerizable thiophene precursors [21]. Using such thiol chemistry, we synthesized the 4-MMDPA tag and covalently attached it to naphthalene-2-thiol through disulfide bonding which is seen as a requirement for the synthesis of many biologically active compounds involved in chemical and biological processes [22]. Diamagnetic and paramagnetic lanthanide ions were then applied to compound ***(10)*** to induce RDC measurements in organic molecules.

## Materials and methods

Benzotriazole, Dimethyl 2,6-pyridinedicarboxylate, 5,5ʹ-dithiobis(2-nitrobenzoic acid), Naphtalene-2-thiol and pyridine dicarboxylic acid were purchased from Sigma Aldrich Chemie GmbH (Schnelldorf, Germany). All lanthanide compounds were purchased from Merck Chemicals GmbH (Darmstadt, Germany). Deuterated solvents were purchased from Deutero GmbH (Kastellaun, Germany). All NMR measurements were recorded at 25 °C on a BRUKER DRX 400 spectrometer and were referenced internally to TMS. The measured RDCs were obtained from the ^13^C dimension of the 2D ^1^H^−13^C–HSQC spectra recorded without carbon decoupling and with a digital resolution of 0.4 Hz/data point. Electron Ionization Mode (EI–HRMS) analysis was conducted on Thermo Fischer Scientific 8230 Finnigan MAT Double Focusing Mass spectrometer with BE Geometry and EI mode at 70 eV (Bremen, Germany).

### Mono-hydroxymethylation

Synthesis of 2,6-dimethoxy carbonyl -4-hydroxyl methylpyridine ***(2)***–Compound ***(1)*** was first identified by ^1^H NMR (400 MHz, CDCl_3_) δ 8.32 (d, *J* = 7.87 Hz, 2H); δ 8.05 (t, *J* = 7.84 Hz, 1H); δ 4.01 (s, 6H), melting point 121 °C. Compound ***(1)*** (2.226 g, 11.4 mmol) was added to a solution containing sulfuric acid (30% v/v) and methanol (50 ml). A saturated aqueous solution of FeSO_4_.7H_2_O was added dropwise followed by H_2_O_2_ solution (30% v/v, 131 mmol) with temperature control between 42 and 45 °C. The reaction was stirred for an additional hour at room temperature and the solution was pH 6.4. The suspension was filtered and extracted with ethyl acetate. The organic phase was dried, filtered, and evaporated to obtain product ***(2)***. After column chromatography (SiO_2_, 100% ethyl acetate), product ***(2)*** (0.665 g, yield: 26%) was identified by ^1^H NMR (400 MHz, CDCl_3_): δ 8.31 (s, 2H); δ 4.91 (s, 2H); δ 4.01 (s, 6H). This reaction was repeated while varying the concentrations of H_2_O_2_ and H_2_SO_4_ and at different temperatures. The optimal conditions are the ones mentioned above.

### Tosylation

Synthesis of 2,6–dimethoxy carbonyl –4–tosyl oxymethylpyridine ***(3)***–A solution of p-toluene sulfonyl chloride (0.4 g, 1.51 mmol) and dichloromethane was added dropwise to a cold solution containing product ***(2)*** (0.665 g, 2.96 mmol) in dichloromethane. Under stirring at 0 °C, triethylamine (0.8 ml) was added dropwise in three portions at 30 min interval. The mixture was diluted with ethyl acetate and the organic phase was extracted with water and 3 M hydrochloric acid. The tosylated product ***(3)*** (0.576 g, yield: 86.5%) was identified by ^1^H NMR (400 MHz, CDCl_3_): δ 8.17 (s, 2H); δ 7.81 (d, *J* = 8.31 Hz, 2H); 2H); δ 7.36 (d, *J* = 8.24 Hz, 2H); δ 5.19 (s, 2H); δ 4.01 (s, 6H); δ 2.45 (s, 3H).

### Bromination

Synthesis of 2,6–Dimethoxy carbonyl –4–bromo methylpyridine ***(4)***–Lithium bromide (0.400 g, 4.60 mmol) was added to a solution containing product ***(3)*** (0.576 g, 1.524 mmol). The solution was stirred at room temperature for 3 h and the suspension filtered, concentrated under vacuum and the residues triturated with chloroform. The product was dissolved in ethyl acetate and purified by preparative thin layer chromatography to obtain the brominated compound ***(4)*** (0.374 g, yield: 65%). ^1^H NMR (400 MHz, CDCl_3_): δ 8.32 (s, 2H); δ 4.50 (s, 2H); δ 4.03 (s, 6H), melting point 114 °C.

### Thiolation

Synthesis of 4–Mercaptomethyl–2,6–pyridinecarboxylic acid ***(6)***–Under nitrogen atmosphere, a solution of ***(4)*** (0.120 g, 0.418 mmol) and thiourea (0.060 g, 0.788 mmol) in methanol was refluxed overnight. The exchange of the bromine by the thiol functional group yielded product ***(5)***, which was then dissolved in deoxygenated water and sodium hydroxide. The mixture was refluxed at 100 °C for 12 h under nitrogen atmosphere. Product ***(6)*** was obtained (0.07 g, 58.3%) ^1^H NMR (400 MHz, D_2_O): δ 8.28 (s, 2H); δ 3.86 (s, 2H), melting point 140 °C, EI–HRMS C_8_H_7_NO_4_S M–H 212.0016.

### Disulfide bridging

Synthesis of 4-(Naphtalen-2-yldisulfanyl)-pyridine-2,6,-dicarboxylic acid ***(10)***–5,5ʹ-Dithio-Bis-(2-Nitrobenzoic acid) (DTNB) ***(8)*** was identified by ^1^H NMR (400 MHz, D_2_O) δ 7.97 (d, *J* = 8.76 Hz, 2H); δ 7.59 (d, *J* = 8.92 Hz, 2H); δ 7.53 (m, 2H). ***(8)*** (1 g, 2.53 mmol) was dissolved in 1:1 methanol/H_2_O solution. Naphtalene-2-thiol ***(7)*** (0.4 g, 2.49 mmol) was added dropwise into DTNB solution. ***(6)*** (0.02 g, 0.094 mmol) was poured over a solution containing ***(7)*** and ***(8)***. The resulting compound (0.3 g, 76.2%) was concentrated, extracted with diethyl ether an identified by ^1^H NMR spectroscopy as compound ***(10)***.

### Incorporation of lanthanide ion

A solution of the tagged molecule ***(10)*** (1.67 mg) was dissolved in DMSO and different lanthanide ions were added as follows: LaCl_3_.7H_2_O (3.7 mg) in 1:1 ratio (10 mM); Eu(FOD)_3_ (2.068 mg) in 0.2:1 ratio (2 mM); Eu_2_(SO_4_)_3_ (1.184 mg) in 0.2:1 ratio (2 mM); Eu(FOD)_3_ (5.17 mg) in 0.5:1 ratio (5 mM); Eu_2_(SO_4_)_3_ (2.959 mg) in 0.5:1 ratio (5 mM); Eu_2_(SO_4_)_3_ (5.918 mg) in 1:1 ratio (10 mM); Tb(NO_3_)_3_ (3.809 mg) in 0.5:1 ratio (10 mM).

## Results and discussion

**Fig. 2:**
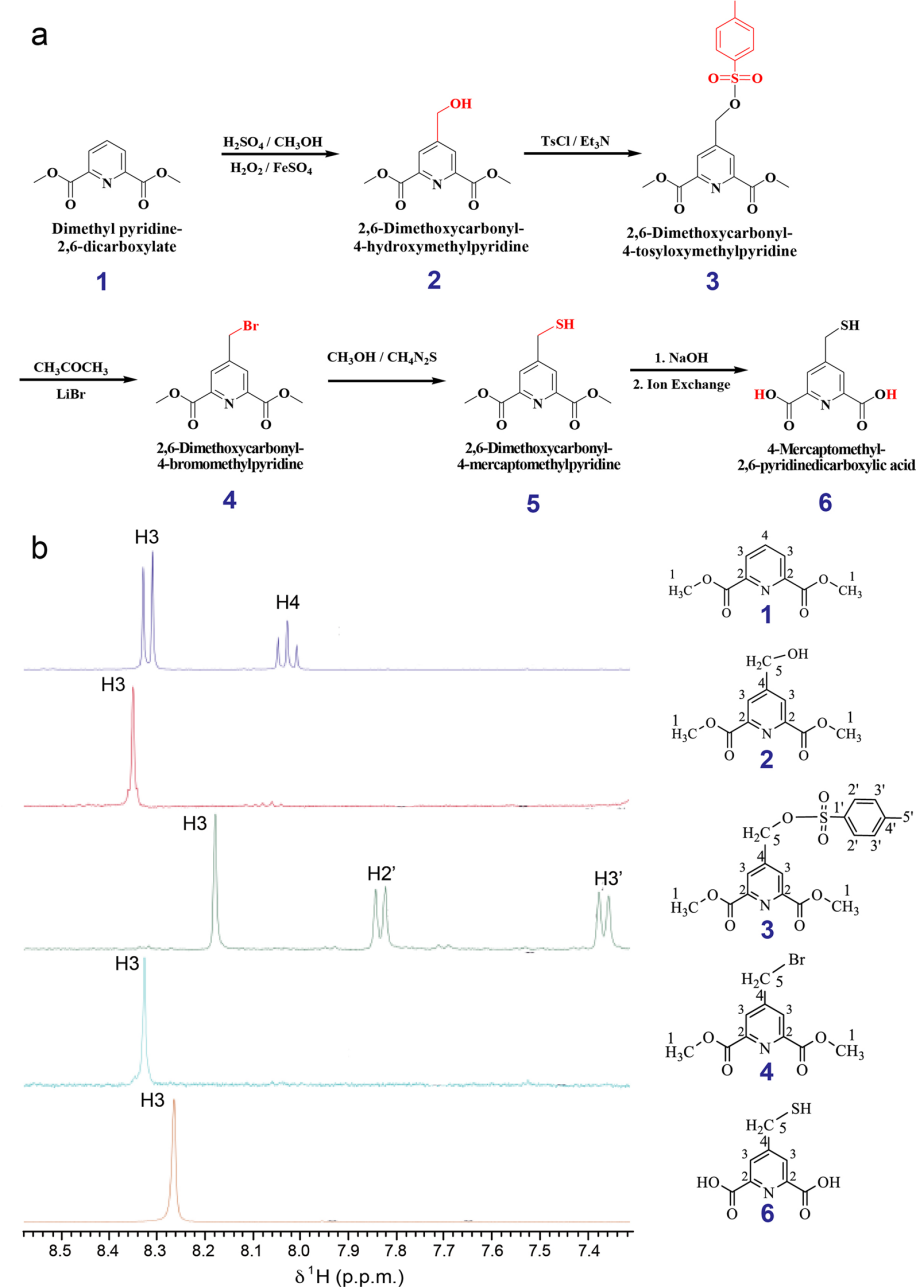
Strategy for the synthesis of 4-MMDPA. **a** synthesis of 4-MMDPA (**b**) 1D ^1^H NMR spectra showing the aromatic region of the intermediates generated along the pathway of 4-MMDPA synthesis. The full spectra of all intermediates is reported in Additional file 1: Figure S1. The color labeling of atoms highlights the substitution of certain functional groups in every step

**Fig. 3:**
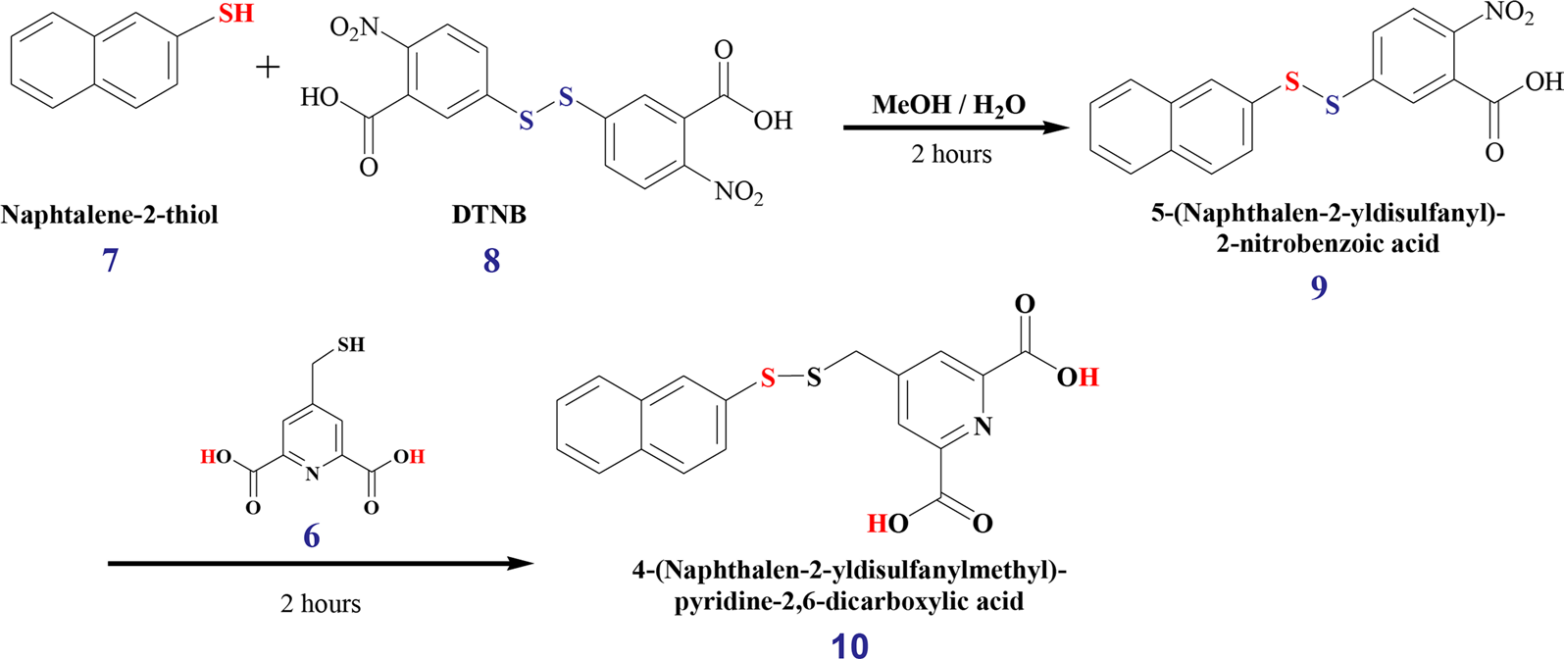
Synthesis of the unsymmetrical disulfide between 4-MMDPA and naphthalene-2-thiol using DTNB chemistry. The color labeling of atoms highlights the substitution of certain functional groups in every step

The design of functional lanthanide-containing coordination compounds requires the precise control of the lanthanide inner coordination sphere [23]. We propose a strategy for measuring RDCs in organic compounds using paramagnetic lanthanide tags. The synthesis of 4-MMDPA ***(6)*** is outlined in Fig. 2 based on modified literature methods [16, 24]. The starting material Dimethyl 2,6-pyridinedicarboxylate ***(1)*** undergoes a direct nucleophilic aromatic substitution to incorporate a mono-hydroxymethyl group ***(2)***, which is then protected by tosylation ***(3)***. The tosyl group is by nature electron-withdrawing hence readily substituted by bromine. The resulting bromomethyl pyridine ***(4)*** undergoes thiolation and generates product ***(5)*** which upon deprotection of its carboxylic moieties yields the desired product 4-MMDPA ***(6)***. All intermediate products were identified by 1D ^1^H NMR spectroscopy (Fig. 2 and Additional file 1: Figure S2) and chemical shift values were accordingly assigned (Table 1). The above reactions were successful after optimizing different conditions. For instance, the temperature control of the mono hydroxymethylation of the starting material ***(1)*** to yield intermediate ***(2)*** was most challenging. At 10 °C, no appreciable conversion of ***(1)*** to ***(2)*** was detected, but after increasing the reaction temperature up to 48 °C, a peak rise was observed (singlet at 8.32 ppm), between the doublet of the starting material, thus indicating the production of the desired intermediate ***(2)*** (Additional file 1: Figure S3). At 50 °C, this desirable intermediate is the major product of the incomplete reaction and shows a sharp resolved singlet between the doublet of the residual starting material. However, the appearance of a new peak on the right side of the doublet (8.29 ppm) indicates the production of an undesirable by-product; which indeed is pronounced in higher yields as the temperature is beyond 50 °C; accompanied by the disappearance of the desired product and its singlet peak at 8.32. Most of the methods developed for the synthesis of sulfur compounds with unsymmetrical S–S bonds, known as unsymmetrical disulfides, involve nucleophilic substitution of a sulfonyl intermediate by a thiol or its derivative [25, 26]. However, preparation of the sulfonyl intermediate requires several steps and the use of toxic chlorinating agents such as SOCl_2_ and Cl_2_. Our attempt to prepare the unsymmetrical disulfide compound from the corresponding thiols, 4-MMDPA ***(6)*** and naphthalene–2–thiol ***(7)***, followed a modified approach with milder conditions that was adopted from disulfide bridging methods of protein chemistry. *5,5ʹ–*Dithiobis (2–nitrobenzoic acid), DTNB, also referred to as Ellman´s reagent, is a symmetrical disulfide commonly used in analytical biochemistry, particularly in labeling cysteine residues. 4-MMDPA can be readily attached to a cysteine thiol group of a protein via a disulfide bridge using established DTNB chemistry [27]. DTNB breaks its disulfide bridge to form the thiol nitrobenzoic acid, which forms a disulfide bond to a thiol–containing compound. We have therefore depicted this method to couple naphthalene–2–thiol ***(7)*** with 4-MMDPA ***(6)*** (synthesis scheme reported in Fig. 3 and ^1^H NMR spectra reported in Additional file 1: Figure S4). DTNB ***(8)*** reacts with naphthalene–2–thiol ***(7)*** to generate the disulfide intermediate product ***(9)***. 4-MMDPA ***(6)*** readily exchanges the TNB moiety of ***(9)*** to yield the desired unsymmetrical disulfide product ***(10)***.

**Table 1:** ^1^H NMR chemical shifts of the intermediate products in the synthesis of 4-MMDPA

Product	Structure	^1^H NMR
*Dimethyl pyridine-2,6-dicarboxylate*		8.32 (d, 2H, H_3_) 8.05 (t, 1H, H_4_) 4.01 (s, 6H, H_1_)
*2,6-Dimethoxycarbonyl-4-hydroxymethylpyridine*		8.31 (s, 2H, H_3_) 4.91 (s, 2H, H_5_) 4.01 (s, 6H, H_1_)
*2,6-Dimethoxycarbonyl-* *4-tosyloxymethylpyridine*		8.17 (s, 2H, H_3_) 7.81 (d, 2H, H_2’_) 7.36 (d, 2H, H_3’_) 5.19 (s, 2H, H_5_) 4.01 (s, 6H, H_1_) 2.45 (s, 3H, H_5’_)
*2,6-Dimethoxycarbonyl-* *4-bromomethylpyridine*		8.32 (s, 2H, H_3_) 4.50 (s, 2H, H_5_) 4.03 (s, 6H, H_1_)
*4-Mercaptomethyl-* *2,6-pyridinedicarboxylic acid*		8.28 (s, 2H, H_3_) 3.86 (s, 2H, H_5_)

Paramagnetic lanthanides are capable of orienting the target molecule in high magnetic fields where each lanthanide orients according to its anisotropic magnetic susceptibility tensor [28, 29]. The principle states that when a paramagnetic lanthanide ion is introduced to a solution, a series of shifted resonances appear in the ^1^H spectrum far outside the spectral range. But with an appropriate order of alignment, RDC measurements are obtained within spectral range from the differences of the paramagnetic and diamagnetic states. The paramagnetic equivalents of scalar couplings, *the contact shifts,* are restricted to nuclei in the immediate vicinity of lanthanides. This induces pseudocontact shifts (PCSs), paramagnetic relaxation enhancements (PREs), and RDCs as the three major effects caused by paramagnetic lanthanide ions in the NMR spectra. Paramagnetic lanthanides provide long–range structural restraints where *PCS* can reach 40 Å from the metal ion and *RDCs* pertain to the entire molecular system due to paramagnetically induced alignment in the magnetic field [13, 30, 31]. Lanthanide ions behave differently when placed in the same chemical environment. This results in different magnitudes of the magnetic susceptibility tensor and its associated anisotropy [32, 33]. Consequently, the lanthanides are ranked according to the magnitude of this anisotropic component as highly paramagnetic (Dy^3+^, Tb^3+^ and Tm^3+^), moderately paramagnetic (Er^3+^ and Yb^3+^), and little paramagnetic (Eu^3+^, Ce^3+^and Sm^3+^). Importantly, there are also diamagnetic lanthanides such as La^3+^, Y^3+^, Lu^3+^ and Sc^3+^ that serve as diamagnetic references. A diamagnetic reference presents the target molecule–metal complex in its diamagnetic state and allows *RD’s* measurements from the differences of the paramagnetic and diamagnetic states. The diamagnetic properties and chemical shifts in particular are sensitive to metal binding [30] nevertheless, the molecule of interest must be site-specifically labeled with a lanthanide in order to fully exploit the lanthanide paramagnetism.

**Fig. 4:**
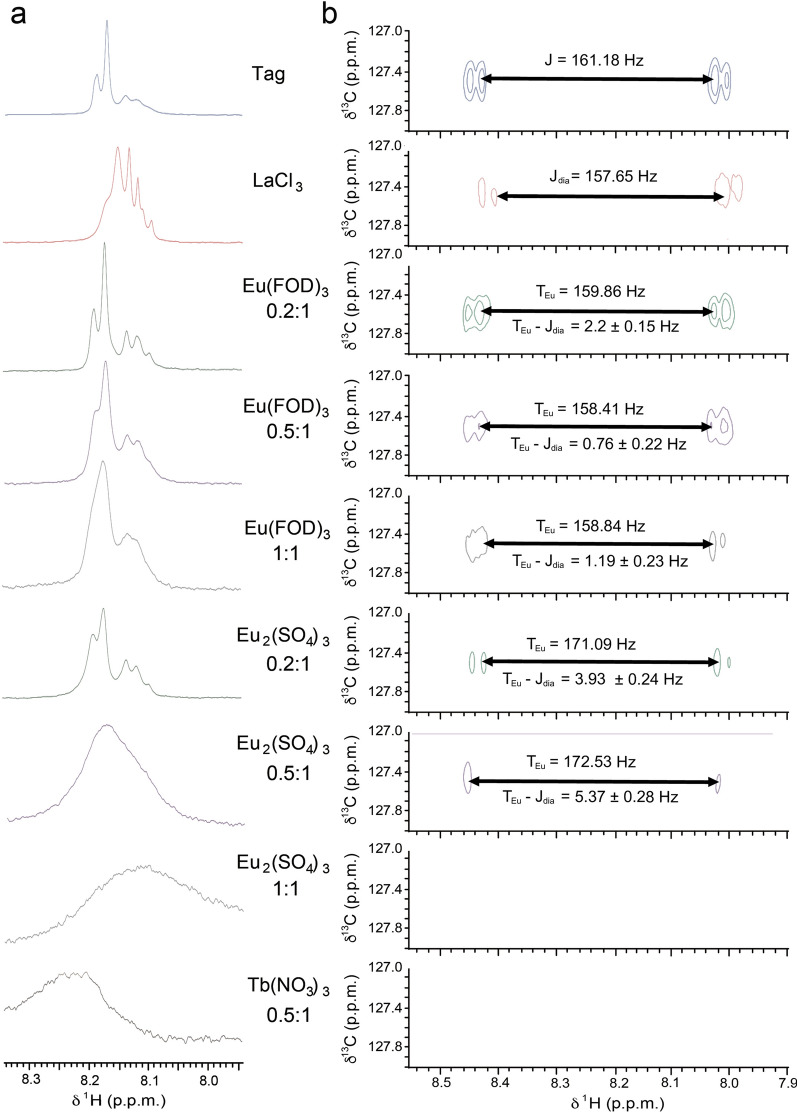
RDC Measurements in Lanthanide tagged 4-MMDPA naphthalene-2-thiol. **a** 1D ^1^H NMR and (**b**) 2D ^1^H-^13^C HSQC spectra of the free tag, and in complex with the diamagnetic lanthanide LaCl_3_, or the paramagnetic lanthanides Eu(FOD)_3_, Eu_2_(SO_4_)_3_, Tb(NO_3_)_3_. The J coupling constants and total coupling constants T for all complexes are shown on the 2D spectra. The error ranges in RDC measurements are evaluated from the contour levels and the peak intensity. H7 of Naphthalene thiol is reported in the spectra

We have investigated the effect of paramagnetic lanthanides by selecting four lanthanide reagents: (a) Lanthanum chloride heptahydrate (LaCl_3_.7H_2_O) as a diamagnetic reference; (b) Europium as a moderately paramagnetic lanthanide ion in its organic state, Eu(FOD)_3_ (tris (6,6,7,7,8,8,8–heptafluoro–2,2–dimethyl–3,5–octanedionato) europium consisting of three bidentate acetylacetonato ligands bound to Eu^3+^ center, and inorganic state (Eu_2_(SO_4_)_3_) and (c) Terbium (III) nitrate pentahydrate (Tb(NO_3_)_3_.5H_2_O) for its highly paramagnetic effect. ^1^H NMR and ^13^C HSQC experiments were first recorded for the diamagnetic reference (1:1 Tag–LaCl_3_.7H_2_O solution, 10 mM) The ^1^H NMR spectra (Fig. 4) showed chemical shifts and peak broadening. A difference in the *J* scalar coupling of the C–H bond of the naphthalene moiety of 3.53 Hz was observed in the ^1^H-^13^C HSQC set as *J*_*dia*_, the scalar coupling from the diamagnetic reference. It is worth mentioning a diamagnetic reference, in this case La^3+^, represents the target molecule–metal complex in its diamagnetic state and allows accurate *RDCs* measurements from the differences of the paramagnetic and diamagnetic states. The diamagnetic properties and chemical shifts are sensitive to metal binding, nevertheless, the molecule of interest must be site-specifically labeled with a lanthanide to fully exploit the lanthanide paramagnetism. The splitting of 3.5 Hz is a clear indication to why it is important to use a diamagnetic reference to report the total coupling rather than using the free tag as a reference. The paramagnetic compound induces shifts in the protons near the Lewis basic sites of the molecule. We measured 1D ^1^H and 2D ^13^C HSQC experiments on three samples of the lanthanide tag (10 mM) while varying Eu(FOD)_3_ concentrations (2 mM, 5 mM and 10 mM). The proton ^1^H spectra (Fig. 4) showed broadening of the peaks and loss of resolution with increasing concentration accompanied with changes in the value of the *total coupling T*. The differences between *T* and *J*_*dia*_ correlate directly to the RDCs values. Indeed, the differences were significant (between 0.76 and 2.21 Hz). Similarly, we measured 1D ^1^H and 2D ^13^C HSQC experiments of the lanthanide tag (10 mM) while varying concentrations of Eu_2_(SO_4_)_3_ (2 mM, 5 mM, and 10 mM). The proton ^1^H spectra (Fig. 4) again showed dramatic peak broadening and loss of resolution as a function of lanthanide concentration. However, the broadening is more significant than in the Eu(FOD)_3_ measurements since the sulfate ions behave differently in solution than the organic ligand, and the Eu ions are more readily available to induce paramagnetism. Similarly, ^13^C HSQC experiments (Fig. 4) showed again significant changes in the value of the total coupling *T*. The differences between *T* and *J*_*dia*_ in the case of Eu_2_(SO_4_)_3_ (3.93 and 5.37 Hz) were larger than the values obtained with Eu(FOD)_3_. All RDCs extracted from the 2D ^1^H-^13^C HSQC spectra of the free tag, and in complex with the diamagnetic lanthanide LaCl_3_, or the paramagnetic lanthanides Eu(FOD)_3_, Eu_2_(SO_4_)_3_, Tb(NO_3_)_3_ are summarized in Additional file 1: Figure S5. RDCs are based on J coupling constants and total coupling constants T for all complexes and include the analysis of errors.

Moreover, the highest lanthanide concentration induced a strong paramagnetic interaction which resulted in peak broadening beyond detection. The ^1^H NMR spectrum of the lanthanide tag in presence of Tb(NO_3_)_3_ (10 mM) proved the strong paramagnetic effect on peak broadening and dramatic loss in resolution. Upon inspection of 1D ^1^H NMR spectra of the 4-MMDPA-naphthalene 2-thiol with different lanthanide concentrations (Fig. 4), the change in intensity demonstrated that there was partial binding of the lanthanide by the time one molar equivalent had been reached in the titration. This made it possible to confer partial alignment and measure small RDCs. The choice of a paramagnetic ion with a higher magnetic susceptibility tensor anisotropy (Tb^3+^) induced a higher degree of alignment upon binding as reflected by signal broadening. It is important to mention that the extraction of accurate and small proton–carbon coupling constants is often complicated and made more difficult by the low natural abundance of ^13^C and spectral overlap. While large one bond proton–carbon coupling constants (^1^*J*_HC_) can be determined easily from ^13^C satellites, *J* values between 0 and 15 Hz are typically much more difficult to be measured and complicated by the similar magnitude of proton–proton and proton–carbon coupling constants [34, 35]. To overcome this, the introduction of H-C coupling in the directly detected dimension by excluding carbon decoupling is a valid approach, rather than in the indirect dimension and omitting the 1H refocusing pi-pulse, might solve spectral overlap but at a cost of decreasing the overall signal to noise ratio.

4-MMDPA coordinates metal ions in a nonchiral fashion and is readily attached to a cysteine thiol group via a disulfide bridge. Lanthanides bind DPA with nanomolar affinity for the first DPA ligand with decreasing affinities for the additional ligands up to the complex [Ln(DPA)_3_]^3–^ [36, 37]. In presence of the lanthanide ion, a target molecule tagged to one unit of 4-MMDPA can further strengthen its coordination using carboxyl groups, if available, and act as additional anchors for tethering the lanthanide ion. Simultaneous coordination of the lanthanide ion by 4–MMDPA and one or several carboxyl groups provides high–affinity lanthanide binding sites that immobilize the lanthanide ion with respect to the target molecule. Importantly, the lanthanide ion must be rigidly attached to the target molecule to avoid averaging due to tag mobility since RDCs are greatly reduced when lanthanide tag reorientates with respect to the protein [38, 39]. This rigidity attachment is accomplished either by tethering the lanthanide tag simultaneously to two different attachment sites of the target molecule [40–42] or by using a bulky lanthanide tag for which reorientation is prevented by steric hindrance [39, 43]. However, lanthanide tags that are anchored to two different sites, such as the case of a protein via two disulfide bonds, immobilize the tag effectively with respect to the protein. Yet, this requires two cysteine residues with thiol groups positioned at the correct distance to react with a single tag molecule [44, 45].

Our attempt to determine the alignment tensor of the 4-MMDPA naphthalene-2-thiol system was unsuccessful due to signal overlap and broadening of all spin pairs other than 1H-C. It is accepted that the size of the observed *RDCs* is directly proportional to the degree of order induced by the anisotropic medium. This degree of orientation should be small otherwise the spectra become too complex to be interpreted. It is indeed essential to maintain both the weak alignment region, where *D* is smaller than *J*, and the quality of the high-resolution NMR spectra so that dipolar coupling constants can be measured easily by comparing line splitting in isotropic and in the aligned samples. However, due to signals overlap of H2, H3, H4, H5 and H6 in the spectrum measured at 400 MHz and signal broadening after the addition of a lanthanide ion, we were limited in using H7 for unambiguous determination of the RDCs. Although this approach proves the concept, a higher magnetic field spectrometer will enhance the quality of the spectra and allow the use of more H-C spin pairs in the analysis.

On the other hand, the single covalent bond between the tag and the thiol group of the target molecule may trigger some flexibility. The presence of such conformational flexibility develops serious consequences in data interpretation and with the structure determination approaches used for rigid compounds as they are inapplicable to flexible compounds. This is due to the simultaneous observation of two averaging processes one from the tumbling motion of the compound and the second due to conformational flexibility. The outcome is an averaging of distances and angles, thus making their use much more complicated. So far, few applications of *RDCs* in flexible molecules have been published; assuming a restricted number of conformers to be present [46, 47].

Metal ions behave as chiral centers such that enantiomers of the metal chelate result in diastereomeric protein-tag complexes, thereby doubling the number of NMR peaks [48]. As a result, the tags’ enantiomeric purity prevents peak doubling caused by metal coordination [49]. Moreover, the proximity of the metal ion to the protein is also significant, because accurate measurement of the alignment tensor A necessitates appropriate sampling of the region around the metal ion [50]. Interactions with the alignment media change the structure of the macromolecule and, as a result, affect the RDCs, therefore alignment by paramagnetic lanthanides rather than alignment media is potentially appealing. Furthermore, lanthanide tags placed at different places on the molecule, or several tags placed at the same region, conveniently offer varied alignment orientations, which are crucial for thorough structural and dynamics research [51].

Paramagnetism causes line broadening for nuclear spins close to the metal ion, making it difficult to measure small RDCs for such nuclei. However, the alignment is independent of the molecule’s molecular weight, implying that a single lanthanide ion might be capable of aligning massive particles. Even though diamagnetic susceptibility grows with molecular size and may exceed paramagnetic susceptibility, an experiment omitting the diamagnetic reference effects would reflect the paramagnetic component of molecular alignment.

## Conclusion

The application of lanthanide tags in biomolecular NMR is a well-established approach to refine structures of proteins complexes by using restraints obtained from paramagnetic NMR experiments. Site–specific labelling of a macromolecule with a lanthanide ion (Ln^3+^) provides access to paramagnetic effects containing long-range structural information of larger proteins and protein–ligand complexes with greater ease and accuracy. However, a largely unexplored method in small molecule structural elucidation is the use of paramagnetic lanthanides to induce RDCs for the determination of relative and absolute configurations.

The specificity of lanthanide labeling is most often achieved by tags that form a covalent bond with a thiol group of the target molecule. The derivatization of thiol groups with lanthanide tags can be achieved easily and in high yields but is specific only if a single thiol group is accessible in the target molecule. In this study, we propose the application of a chiral paramagnetic lanthanide tag (4-MMDPA) to induce partial alignment in a target molecule (naphthalene-2-thiol) and measure RDCs. A diamagnetic reference (LaCl_3_) is compared with different paramagnetic lanthanides (Eu(FOD)_3_, Eu_2_(SO_4_)_3_ and Tb(NO_3_)_3_). Once a proper alignment within the magnetic field is achieved, the paramagnetic solution shows total coupling constants, *T*, and the differences with the scalar couplings from the diamagnetic solution, *J*, would mark the RDC constants, *D*. This method allows structural determination of small molecules with accurate molecular conformation and configuration information. The lanthanide tag method is widely used in protein structural determination, limited research has been conducted with small molecules due to the broadening effect. This strategy broadens the application of paramagnetic lanthanide tags for the measurement of RDCs in organic molecules.

## Supplementary Information

**Additional file 1:** **Figure S1.** Residual Dipolar Couplings in NMR Spectroscopy. Dipolar interaction between spins *I *and *S *(a) parallel and (b) anti-parallel to the magnetic field B0. (c) Dipolar coupling splitting 2DIS relative to B0. (d) Representation of the probability tensor *P *in the molecular frame reference system as depicted from Kramer et al. (*Concepts in Magnetic Resonance Part A *2004). **Figure S2.** Synthesis of 4-MMDPA followed by NMR Spectroscopy (a) 1D 1H NMR spectra showing the aromatic region of the intermediates generated along the pathway of 4-MMDPA synthesis. **Figure S3.** Optimization of the monohydroxy methylation step. 1H NMR spectra showing the temperature effect on the monohydroxymethylation reaction of the dimethyl pyridine-2,6, dicarboxylate. **Figure S4.** 1H NMR spectra showing the starting material 4-MMDPA and the 2- naphthalene thiol prior bonding to the lanthanide tagging.** Figure S5.** Representation of the RDCs extracted from the 2D 1H-13C HSQC spectra of the free tag, and in complex with the diamagnetic lanthanide LaCl3, or the paramagnetic lanthanides Eu(FOD)3, Eu2(SO4)3, Tb(NO3)3. RDCs are based on J coupling constants and total coupling constants T for all complexes and include the analysis of errors.

## Data Availability

All data generated or analyzed during this study are included in this published article and its Additional file 1. The NMR chemical shift of 4-MMDPA has been deposited in bmrbig database (https://bmrbig.org) under the accession number BMRbig42 (https://bmrbig.org/released/bmrbig42).

## Abbreviations

NMR: Nuclear magnetic resonance
4-MMDPA: 4-Mercaptomethyl-dipicolinic acid
RDC: Residual dipolar coupling
