# Supplementary material for: Synthesis of lanthanide tag and experimental studies on paramagnetically induced residual dipolar couplings
Source: BMC Chem. 2022 Jul 21;16(1):54. doi: 10.1186/s13065-022-00847-5 (PMC9306141; doi:10.1186/s13065-022-00847-5)
Supplement: Supplementary file 1 — Additional file 1: Figure S1. Residual Dipolar Couplings in NMR Spectroscopy. Dipolar interaction between spins I and S (a) parallel and (b) anti-parallel to the magnetic field B0. (c) Dipolar coupling splitting 2DIS relative to B0. (d) Representation of the probability tensor P in the molecular frame reference system as depicted from Kramer et al. (Concepts in Magnetic Resonance Part A 2004). Figure S2. Synthesis of 4-MMDPA followed by NMR Spectroscopy (a) 1D 1H NMR spectra showing the aromatic region of the intermediates generated along the pathway of 4-MMDPA synthesis. Figure S3. Optimization of the monohydroxy methylation step. 1H NMR spectra showing the temperature effect on the monohydroxymethylation reaction of the dimethyl pyridine-2,6, dicarboxylate. Figure S4. 1H NMR spectra showing the starting material 4-MMDPA and the 2- naphthalene thiol prior bonding to the lanthanide tagging. Figure S5. Representation of the RDCs extracted from the 2D 1H-13C HSQC spectra of the free tag, and in complex with the diamagnetic lanthanide LaCl3, or the paramagnetic lanthanides Eu(FOD)3, Eu2(SO4)3, Tb(NO3)3. RDCs are based on J coupling constants and total coupling constants T for all complexes and include the analysis of errors. [file 13065_2022_847_MOESM1_ESM.pdf]

Synthesis of Lanthanide Tag and Experimental Studies on Paramagnetically Induced Residual Dipolar Couplings

Ali Yassin,[1, 2] Bilal Nehmeh,[1] Sally El Kantar,[1,3] Yara Al Kazzaz,[1] Elias Akoury[1,4,*]

[1] Department of Natural Sciences, School of Arts and Sciences, Lebanese American University, Beirut 1102-2801, Lebanon

[2] Inorganic and Organometallic Coordination Chemistry Laboratory, LCIO, Lebanese University, Faculty of Science, Beirut, Lebanon

[3] Université de technologie de Compiègne, ESCOM, TIMR (Integrated Transformations of Renewable Matter), Centre de recherche Royallieu, CS 60 319, 60203 Compiègne Cedex, France

[4] Department of Chemistry, Faculty of Chemistry and Pharmacy, Ludwig Maximilian University, 81377 Munich, Germany
[*] Correspondence to elias.akoury@lau.edu.lb

Supplementary Information

Supplementary text

Using quantum mechanics, the dipolar interaction of nuclear spins rotating at the *Larmor frequency* is described in a simplified two-spin system. Spins ***I*** and ***S*** with a fixed distance $\mathbf{R_{IS}}$ are oriented at an internuclear angle $\boldsymbol{\Theta_{IS}}$ in a static magnetic field $\mathbf{B_0}$ (**Supplementary figures 1a and 1b**). Spin ***I*** induces a magnetic field added to the static magnetic field felt by spin ***S*** and causes a shift in its resonance frequency, the dipolar coupling.[1] Since spins parallel and anti-parallel to $\mathbf{B_0}$ are equally populated, splitting into the dipolar coupling $\boldsymbol{2D_{IS}}$ is observed and is directly proportional to $3\cos^2\Theta_{IS} - 1$ (**Supplementary figure 1c**). This RDC contains valuable structural information and covers a large portion of the NMR timescale allowing its application in small organic molecules[2-3], in biomolecules[4-5] and in newly discovered types of materials.[6-7]

The behavior of a molecule with $\mathbf{B_0}$ differs largely if it is in crystal form, in isotropic solution or in anisotropic solution with a partially oriented medium (**Supplementary figure 1d**). In the latter case, the quantum mechanical approach of RDCs is based on the averaging process of the anisotropic tumbling motion, which occurs between the molecule being subjected to a magnetic field, and is described by the alignment tensor ***A***.[8] The dipolar coupling Hamiltonian $\boldsymbol{H_D}$ depicting the two spins I and S has the form:

$$H_D = 2\pi D\left\{I_{z^L}S_{z^L} - \frac{1}{2}I_{x^L}S_{x^L} - \frac{1}{2}I_{y^L}S_{y^L}\right\} \qquad (1)$$

If the spins *I* and *S* are heteronuclear, $\boldsymbol{H_D}$ is simplified to:

$$H_D = 2\pi D I_{z^L}S_{z^L} \qquad (2)$$

and the dipolar coupling constant ***D*** is represented by the equation:

$$D = \frac{K}{R^3}\left(cos^2\Theta_{IS} - \frac{1}{3}\right) \qquad (3)$$

K is a term that gathers the physical constants of the spins:

$$K = -\frac{3}{8\pi^2}(\gamma_I \gamma_S \mu_0 \hbar) \tag{4}$$

where γ_I and γ_S are the gyromagnetic ratios of spins I and S, respectively, μ_0 is the vacuum permeability, and $\hbar$ is Plank´s constant. It is worth mentioning that ***D*** and ***H**$_D$* are both time-dependent, and therefore, the time-averaged *RDC constant* $\overline{D}$ is represented as:

$$\overline{D} = \frac{K}{R^3}\left(\overline{cos^2\Theta_{IS}} - \frac{1}{3}\right) \tag{5}$$

To calculate $\overline{D}$ for any pair of spins, the alignment properties of a molecule in the molecular frame approach must be first described. In this reference system, the orientation of the magnetic field is time-dependent, the inter-nuclear vector is set constant, and the *probability tensor* ***P*** is introduced. ***P*** is a second order approximation of the orientation probability distribution for the direction of the external magnetic field[9] and is represented by an ellipsoid with fixed orientation in the (x, y, z) molecular frame (**Supplementary Figure 1e**). The three principal values of ***P*** are the probabilities of finding the magnetic field along the principal axes of the ellipsoid such that

$$P_x + P_y + P_z = 1 \tag{6}$$

This simplifies the calculation of the RDC constants and requires only knowledge of the three Cartesian components r_x, r_y and r_z of a given inter-nuclear unit vector $\vec{r}$:

$$\overline{cos^2\Theta_{IS}} = P_x r_{\tilde{x}}^2 + P_y r_{\tilde{y}}^2 + P_z r_{\tilde{z}}^2 \tag{7}$$

The alignment tensor ***A*** of the anisotropic tumbling motion is then extracted from ***P*** and fitted with the principal components:

$$A = P - \frac{1}{3} \tag{8}$$

$$A_x + A_y + A_z = 0 \tag{9}$$

$$\left(\overline{cos^2\Theta_{IS}} - \frac{1}{3}\right) = A_x r_{\tilde{x}}^2 + A_y r_{\tilde{y}}^2 + A_z r_{\tilde{z}}^2 \tag{10}$$

The time-averaged RDC representing the direct dipole-dipole interaction for spins ***I*** and ***S*** is represented by the equation:

$$\overline{D_{IS}} = -\frac{\gamma_I \gamma_S \mu_0 \hbar}{16\pi^2} \langle \frac{1}{R_{IS}^3} \left(3\overline{cos^2\Theta_{IS}} - 1\right) \rangle \qquad (11)$$

The observed anisotropic dipolar coupling $\boldsymbol{D_{IS}}$ adds to the scalar ***J*** coupling, and both contribute to the total coupling constant ***T***:

$$|T| = |J + D| \qquad (12)$$

Therefore, two measurement series are required to determine the dipolar coupling ***D*** for a spin system; an isotropic spectrum that determines ***J*** scalar couplings, and another anisotropic spectrum to measure ***T*** total couplings. The difference between the two spectra determines ***D***.

Supplementary Figures

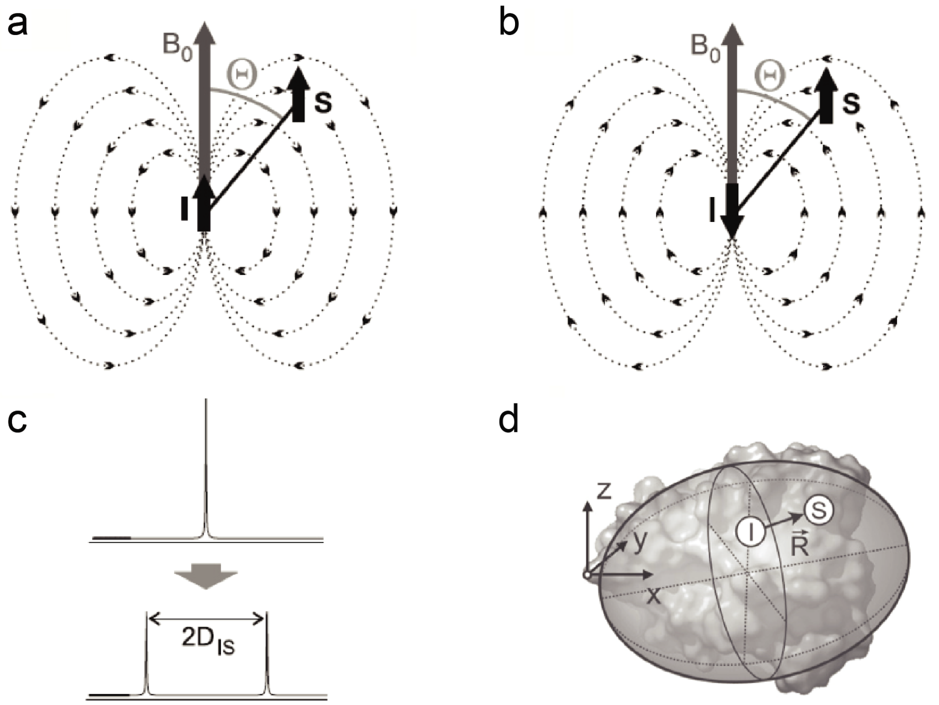

Supplementary Figure 1. Residual Dipolar Couplings in NMR Spectroscopy. Dipolar interaction between spins *I* and *S* (a) parallel and (b) anti-parallel to the magnetic field B_0. (c) Dipolar coupling splitting $2D_{IS}$ relative to B_0. (d) Representation of the probability tensor *P* in the molecular frame reference system as depicted from Kramer et al. (*Concepts in Magnetic Resonance Part A* 2004).[8]

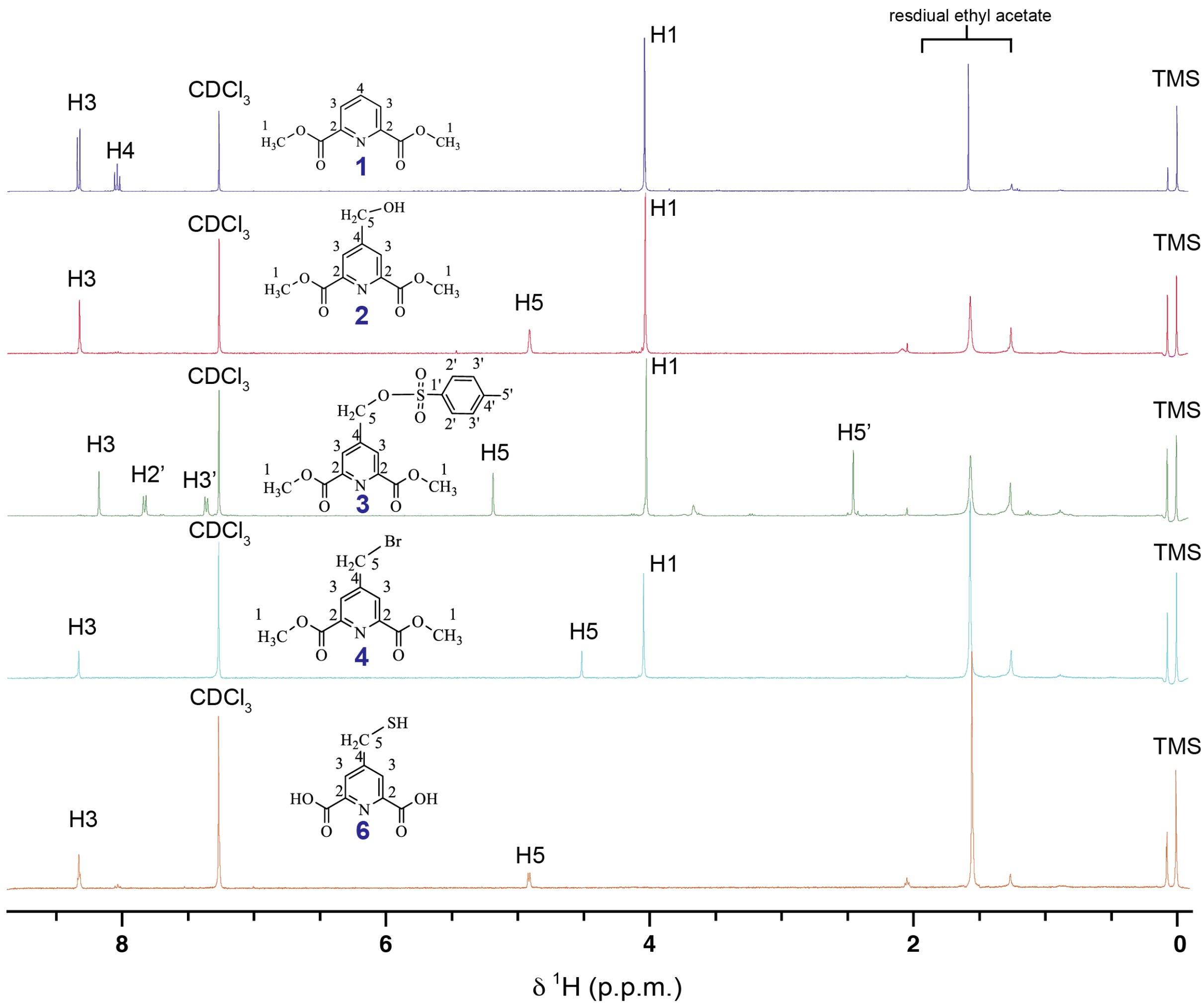

Supplementary Figure 2 – Synthesis of 4-MMDPA followed by NMR Spectroscopy (a) 1D ^{1}H NMR spectra showing the aromatic region of the intermediates generated along the pathway of 4-MMDPA synthesis.

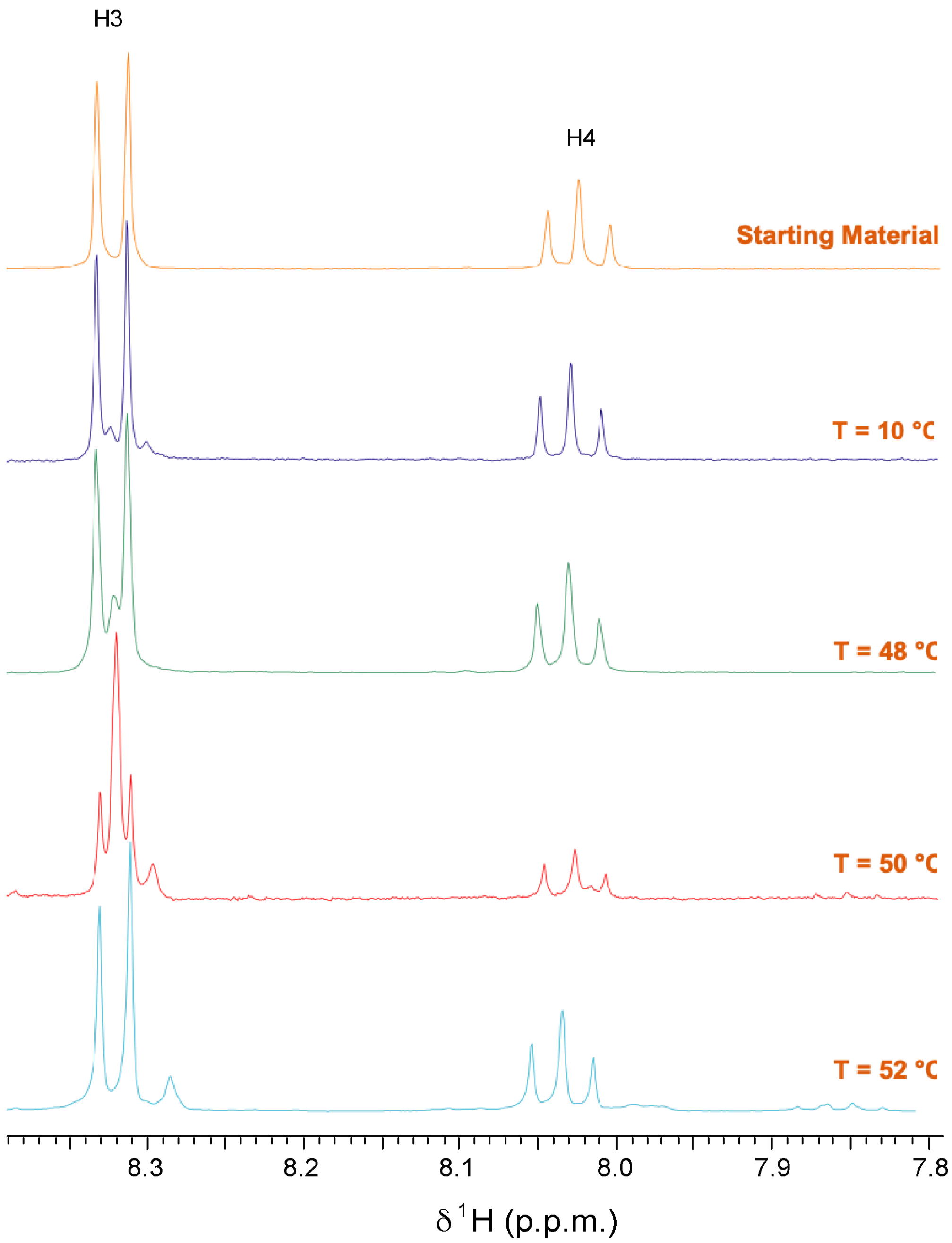

Supplementary Figure 3 – Optimization of the monohydroxy methylation step. ^{1}H NMR spectra showing the temperature effect on the monohydroxymethylation reaction of the dimethyl pyridine-2,6, dicarboxylate.

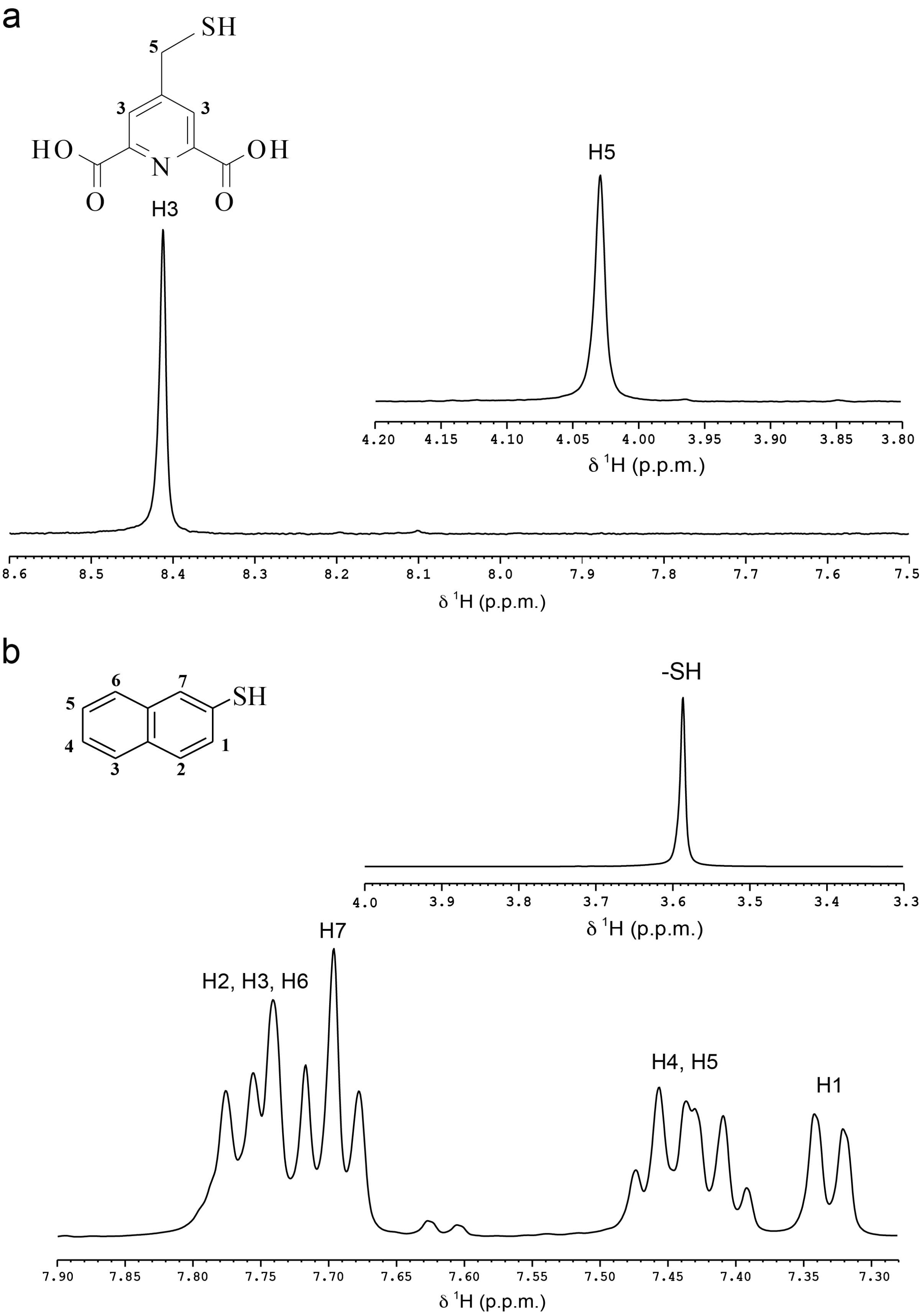

Supplementary Figure 4 – ^{1}H NMR spectra showing the starting material 4-MMDPA and the 2-naphthalene thiol prior bonding to the lanthanide tagging.

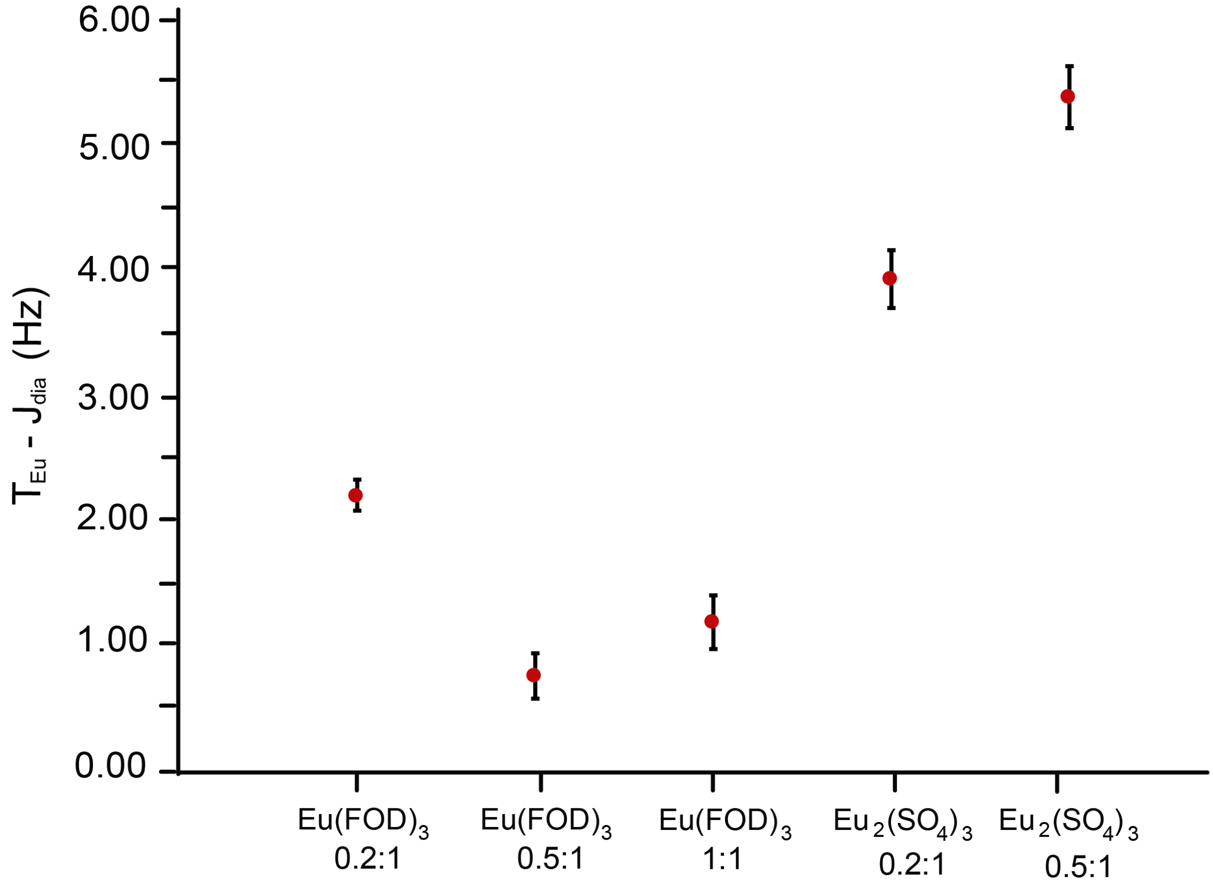

Supplementary Figure 5 – Representation of the RDCs extracted from the 2D ^{1}H-^{13}C HSQC spectra of the free tag, and in complex with the diamagnetic lanthanide $LaCl_3$, or the paramagnetic lanthanides $Eu(FOD)_3$, $Eu_2(SO_4)_3$, $Tb(NO_3)_3$. RDCs are based on J coupling constants and total coupling constants T for all complexes and include the analysis of errors.

1. Kummerlowe, G.; Crone, B.; Kretschmer, M.; Kirsch, S. F.; Luy, B., Residual dipolar couplings as a powerful tool for constitutional analysis: the unexpected formation of tricyclic compounds. *Angew Chem Int Ed Engl* **2011,** *50* (11), 2643-5.
2. Liu, Y.; Navarro-Vazquez, A.; Gil, R. R.; Griesinger, C.; Martin, G. E.; Williamson, R. T., Application of anisotropic NMR parameters to the confirmation of molecular structure. *Nat Protoc* **2019,** *14* (1), 217-247.
3. Ge, H. M.; Sun, H.; Jiang, N.; Qin, Y. H.; Dou, H.; Yan, T.; Hou, Y. Y.; Griesinger, C.; Tan, R. X., Relative and absolute configuration of vatiparol (1 mg): a novel anti-inflammatory polyphenol. *Chemistry* **2012,** *18* (17), 5213-21.
4. Akoury, E.; Ma, G.; Demolin, S.; Bronner, C.; Zocco, M.; Cirilo, A.; Ivic, N.; Halic, M., Disordered region of H3K9 methyltransferase Clr4 binds the nucleosome and contributes to its activity. *Nucleic Acids Res* **2019,** *47* (13), 6726-6736.
5. Akoury, E.; Mukrasch, M. D.; Biernat, J.; Tepper, K.; Ozenne, V.; Mandelkow, E.; Blackledge, M.; Zweckstetter, M., Remodeling of the conformational ensemble of the repeat domain of tau by an aggregation enhancer. *Protein Sci* **2016,** *25* (5), 1010-20.
6. Chedid, G.; Yassin, A., Recent Trends in Covalent and Metal Organic Frameworks for Biomedical Applications. *Nanomaterials (Basel)* **2018,** *8* (11).
7. Riechers, B.; Maes, F.; Akoury, E.; Semin, B.; Gruner, P.; Baret, J. C., Surfactant adsorption kinetics in microfluidics. *Proc Natl Acad Sci U S A* **2016,** *113* (41), 11465-11470.
8. Kramer, F.; Deshmukh, M. V.; Kessler, H.; Glaser, S. J., Residual dipolar coupling constants: An elementary derivation of key equations. *Concepts in Magnetic Resonance Part A* **2004,** *21A* (1), 10-21.
9. Prestegard, J. H.; al-Hashimi, H. M.; Tolman, J. R., NMR structures of biomolecules using field oriented media and residual dipolar couplings. *Q Rev Biophys* **2000,** *33* (4), 371-424.
